# Brain activation is related to smoothness of upper limb movements after stroke

**DOI:** 10.1007/s00221-015-4538-8

**Published:** 2016-03-15

**Authors:** Floor E. Buma, Joost van Kordelaar, Matthijs Raemaekers, Erwin E. H. van Wegen, Nick F. Ramsey, Gert Kwakkel

**Affiliations:** Center of Excellence for Rehabilitation, Rehabilitation Centre De Hoogstraat, Rembrandtkade 10, 3583TM Utrecht, The Netherlands; Department of Neurology and Neurosurgery, Rudolf Magnus Institute of Neuroscience, UMC Utrecht, PO Box 85060, 3508AB Utrecht, The Netherlands; Department of Rehabilitation Medicine, MOVE Research Institute Amsterdam, VU University Medical Center, PO Box 7057, 1007MB Amsterdam, The Netherlands; Amsterdam Rehabilitation Research Center, Reade Centre for Rehabilitation and Rheumatology, PO Box 58271, 1040HG Amsterdam, The Netherlands

**Keywords:** Stroke, Neuroplasticity, Recovery, Upper extremity, Brain activation, Motor control

## Abstract

It is unclear whether additionally recruited sensorimotor areas in the ipsilesional and contralesional hemisphere and the cerebellum can compensate for lost neuronal functions after stroke. The objective of this study was to investigate how increased recruitment of secondary sensorimotor areas is associated with quality of motor control after stroke. In seventeen patients (three females, fourteen males; age: 59.9 ± 12.6 years), cortical activation levels were determined with functional magnetic resonance imaging (fMRI) in 12 regions of interest during a finger flexion–extension task in weeks 6 and 29 after stroke. At the same time points and by using 3D kinematics, the quality of motor control was assessed by smoothness of the grasp aperture during a reach-to-grasp task, quantified by normalized jerk. Ipsilesional premotor cortex, insula and cerebellum, as well as the contralesional supplementary motor area, insula and cerebellum, correlated significantly and positively with the normalized jerk of grasp aperture at week 6 after stroke. A positive trend towards this correlation was observed in week 29. This study suggests that recruitment of secondary motor areas at 6 weeks after stroke is highly associated with increased jerk during reaching and grasping. As jerk represents the change in acceleration, the recruitment of additional sensorimotor areas seems to reflect a type of control in which deviations from an optimal movement pattern are continuously corrected. This relationship suggests that additional recruitment of sensorimotor areas after stroke may not correspond to restitution of motor function, but more likely to adaptive motor learning strategies to compensate for motor impairments.

## Introduction

Outcomes of neurorehabilitation after stroke are variable and depend largely on the intensity and task specificity of the intervention applied as well as the severity of initial impairment at stroke onset (Langhorne et al. 2011). For the paretic upper limb in particular, treatment effects are mainly restricted to patients with some voluntary control of finger extension after stroke (Kwakkel and Kollen 2013; Langhorne et al. 2011). These findings suggest that there is a need for a better understanding of the neuronal mechanisms underlying functional recovery after stroke.

Task-related recruitment of secondary sensorimotor areas in the affected and non-affected hemisphere has been associated with poor motor recovery in terms of body functions and activities (Buma et al. 2010; Ward et al. 2004). It is therefore unlikely that secondary sensorimotor areas are able to take over the functions of the primary injured motor areas (Buma et al. 2010; Ward et al. 2004). Recruitment of these additional areas may rather reflect support in the execution of compensatory motor control while performing a motor task with the paretic upper limb.

However, it is still unclear how brain activation patterns are associated with quality of upper limb control after stroke (Buma et al. 2013). Most traditional clinical assessment scales are not suitable for capturing *how* patients perform functional tasks. By contrast, 3D kinematics can assess intra-limb coordination and smoothness of movement patterns, which are important characteristics of quality of motor control.

A recent study with intensive repeated 3D kinematic measurements in the first 6 months after stroke suggested that basic synergistic couplings between the shoulder and elbow during a functional reaching task diminished as a function of time after stroke (van Kordelaar et al. 2013). This suggests that the ability to plan movements in advance (i.e. feedforward motor control) may improve, thereby decreasing the continuous online corrections based on proprioceptive feedback (van Kordelaar et al. 2014; Meulenbroek et al. 2001). Such corrections based on afferent information have been shown to negatively affect the smoothness of hand and finger movements (Merdler et al. 2013).

An important measure to quantify smoothness is normalized jerk. Jerk is the third time derivative of the position of a particular body part. Normalized jerk is obtained by correcting for differences in movement duration and movement distance (Caimmi et al. 2008). As high smoothness is reflected by minimal changes in position, smoothness is inversely related to normalized jerk. We have recently shown that this jerk measure decreases (i.e. smoothness increases) substantially in the first 8 weeks after stroke (van Kordelaar et al. 2014) and levels off up to 26 weeks after stroke, suggesting that jerkiness is a sensitive measure to investigate time-dependent changes in quality of motor control, particularly early after stroke. However, due to a lack of studies combining imaging techniques with kinematic analyses, the neurological mechanisms underlying the recovery of smoothness of upper limb movements are still largely unknown.

We hypothesized that elevated recruitment of secondary sensorimotor areas would be associated with jerky movements. This hypothesis was tested by investigating the association between smoothness of finger movements during a reach-to-grasp task, measured with 3D kinematics, and activation levels in sensorimotor networks of the brain during a finger flexion–extension task, measured with functional MRI (fMRI) (Buma et al. 2010). There are strong indications that the potential for neural adaptation is mainly limited to a time window of 10 weeks after stroke in which most spontaneous neurological recovery occurs (Murphy and Corbett 2009; Langhorne et al. 2011). We tested the association between brain activation and smoothness of finger movements at 6 and 29 weeks after stroke, to assess whether this association changes with time after stroke (Buma et al. 2010; van Kordelaar et al. 2014).

## Methods

### Patients

Seventeen patients (three females and fourteen males) with stroke were included in this study. Patients had a mean age of 59.9 years (SD = 12.6 years) and were included if they (1) had had their first ever ischaemic stroke and had suffered from mono- or hemiparesis of the hand at the time of their stroke; (2) were between 18 and 80 years old; (3) were able to understand instructions as indicated by a mini-mental state examination (MMSE) score of 23 or higher (Folstein et al. 1975); and (4) gave written consent to participate in the study. Exclusion criteria were (1) not being able to make flexion–extension movements with the fingers or reach-to-grasp movements with the paretic upper limb in week 6 after stroke; (2) pacemakers or other metallic implants incompatible with the 3T MRI scanner; (3) orthopaedic impairments of the upper extremities; (4) communication restrictions as indicated by a score of 3 or less on the Utrecht Communication Observation (UCO) (Schepers et al. 2005); and (5) botulinum toxin injections or other medication influencing the function of the upper limb.

Seventeen patients were recruited within the EXPLICIT-stroke programme, and they were stratified according to the ability to perform some finger extension within 1 week after stroke (Kwakkel et al. 2008). Patients with an unfavourable prognosis based on finger extension were randomly allocated to the experimental group that received electromyography-triggered neuromuscular stimulation (EMG-NMS) or the control group that received usual care (*N* = 5). Patients with a favourable prognosis were randomly allocated to the experimental group that received modified constraint-induced movement therapy (mCIMT) or the control group that received usual care (*N* = 12) (Kwakkel et al. 2008). EMG-NMS and mCIMT were applied from week 2 to week 5 after stroke. Handedness was established with the Edinburgh Handedness Inventory (Oldfield 1971). After the experimental intervention period, all patients who participated in this study underwent two fMRI and two 3D kinematic measurements, performed at weeks 6 and 29 after stroke. To avoid effects of fatigue, measurements were performed on separate days. Informed consent was obtained according to the Declaration of Helsinki, and the study protocol was approved by the local ethics committee.

### Clinical measurements

Motor function of the affected arm of each patient was assessed at 6 and 29 weeks after stroke using the upper extremity section of the Brunnstrom Fugl-Meyer Motor Assessment (FMA), the Action Research Arm Test (ARAT) and the nine-hole peg test (NHPT). The FMA test is an assessment based on the concept of sequential stages of motor recovery (Fugl-Meyer et al. 1975), and it tests reflexes, basic limb synergies of the paretic upper limb and hand function. Each item is scored on an ordinal 3-point scale to express a motor score for the affected side, with a total score ranging from 0 to 66. The ARAT is a clinical test of arm motor function (Lyle 1981). Upper limb movements, in terms of pinch, grasp, grip and gross movements, are performed and scored on a 4-point scale, with a total score ranging from 0 to 57. The NHPT measures dexterity of the hand, focusing on fine motor function. Pegs are inserted and removed from a nine-hole peg board; scores are based on the time (in seconds) taken to complete the test and were calculated as percentage of healthy sample norms (Oxford Grice et al. 2003).

### Functional MRI

#### Data acquisition

Images were acquired with two Philips Achieva 3.0 Tesla MRI scanners (Philips, Eindhoven, Netherlands), located at UMCU and LUMC. Patients recruited from hospitals near Utrecht (*N* = 9) were measured with the scanner at UMCU, and patients recruited near Leiden were measured with the LUMC scanner (*N* = 8). High-resolution whole-brain anatomical scans were acquired for all subjects for anatomical reference (3D T1-weighted scan: TR = 9.717 ms; TE = 4.59 ms, flip angle = 8°, 140 slices, 0.875 × 0.857 × 1.2 mm, FOV = 224 × 168 × 177). During the motor task, 384 fMRI PRESTO scans were acquired (flip angle = 10°, FOV = 224 × 256 × 160 mm, voxel size 4 × 4×4 mm, TE/TR = 33/23 ms, time per whole-brain volume 0.63 s) (Neggers et al. 2008). To check for mirror movements, EMG was applied to the hand opposite the moving hand with four scanner-compatible surface electrodes (MR Physiology Logging, Philips Medical Systems BV, Eindhoven, The Netherlands).

#### Motor paradigm

During the fMRI measurements, flexion–extension of the fingers of the affected hand was paced at 1 Hz (i.e. 1 movement/s) by means of an arrow on a computer screen (alternating 30 s of movement and 30 s of rest for a period of 4 min). In addition, patients wore headphones to minimize the level of perceived noise induced by the MRI scanner. Patients’ hand and wrist were enclosed by a plastic orthosis only allowing simultaneous movement of 4 fingers of the hand flexing only at the MCP joints. Thumb and wrist were restrained as previous studies found extension of the fingers to be one of the most important predictors of functional outcome after stroke (Nijland et al. 2010; Stinear 2010). In addition, the thumb has been shown to be mainly invariant during reach-to-grasp movements, whereas the fingers contributed most to the grasping movement (Galea et al. 2001). During the entire fMRI assessment, both arms rested comfortably alongside the patient’s hips, with the elbows slightly bent in a comfortable position. Task performance was monitored visually by the researcher present during scanning.

#### Data preprocessing for fMRI

FMRI data were analysed with Statistical Parametric Mapping (SPM5) software (http://www.fil.ion.ucl.ac.uk/spm/) in MATLAB (MATLAB 11.1; The Mathworks Inc, MathWorks, Natick, Massachusetts). All functional images of each participant were realigned to the first functional scan of each session. After realignment, all images were co-registered to the T1-weighted anatomical scan. Subsequently, images were transformed to standard Montreal Neurological Institute (MNI) space and smoothed using a Gaussian kernel with a 8-mm full width at half maximum, while also keeping the non-smoothed data. The task boxcar function was convolved with the canonical hemodynamic response function, and the resulting model was estimated in combination with a high-pass filter with a cut-off at 128 s to remove low-frequency artefacts. In the first-level analysis, contrast maps were calculated using a general linear model representing periods of motor activity versus rest for each patient and each session separately (Friston et al. 1995; Worsley and Friston 1995). Contrast images containing the regression coefficients, i.e. beta values, for each voxel from twelve patients with right-sided lesions were flipped across the mid-sagittal plane, so that the affected hemisphere corresponded to the left side of the brain for all patients.

#### ROI data analysis

A region of interest (ROI)-based comparison was performed using the unsmoothed data. An automatic segmentation procedure (Freesurfer ASEG) (Fischl et al. 2004) was applied using the individual anatomical images of each subject to delineate the cortical areas, including the bilateral precentral and postcentral gyrus, supplementary motor area, premotor cortex, cerebellum and insula. All motor segments were visually inspected to ensure correct segmentation for each subject. The volumes containing the motor segments were normalized to MNI space using the previously estimated normalization parameters. ROI activation levels were established by taking the 15 % most active voxels during the motor task in each anatomical motor segment. A proportional rather than an absolute threshold was used in the ROI definition to account for between-subject differences in the volume of activation (Raemaekers et al. 2012). Blood oxygen level-dependent (BOLD) signal changes per ROI were represented by the mean beta value during each task.

#### Detection of potential mirror movements with EMG

The EMG data were analysed as described by Van Rootselaar and colleagues (van Rootselaar et al. 2008). During each fMRI session, the EMG signal was recorded using electrodes attached to the hand contralateral to the moving hand, over the musculus extensor digitorum communis and musculus abductor pollicis brevis. The EMG data were analysed in MATLAB version 2011a. First, the EMG signal was notch-filtered at 45 and 90 Hz to remove fMRI artefacts induced by the gradient magnets and high-pass filtered at 10 Hz to remove movement artefacts. The signal was rectified to regain low-frequency components. Data were then band-pass filtered between 2 and 130 Hz, and a correlation coefficient was calculated for the envelope of the signal time series and the task as a boxcar function. Subjects were asked to extend their hand maximally as a measure of maximal voluntary extension (MVE) before every task in the scanner. The corresponding EMG signal over that time was averaged and used as a norm value for average %MVE during movement blocks. Average %MVE was calculated by dividing the average EMG signal during the task by the average MVE and multiplying this by 100 %. A score for the presence of mirror movements was calculated from the correlation coefficient of the envelope of the EMG signal and the task boxcar, multiplied by the value for %MVE. This score was correlated with the average beta for each contralesional ROI.

### 3D Kinematics

#### Data acquisition

3D kinematic data were collected using a portable electromagnetic motion-tracking device (Polhemus Liberty, Polhemus, Vermont). Motion sensors were attached to the trunk, scapula, upper arm, forearm, hand, thumb and index finger of the paretic upper limb. This study focused on the data obtained from the thumb and index finger sensors. The sampling frequency was 240 Hz. Before each measurement, a pointer device (ST8, Polhemus Liberty, Polhemus, Vermont) was used to locate the tips of the thumb and index finger relative to their associated finger sensors.

Measurements were conducted at the site where patients resided. A previous study showed that data could be accurately and reliably recorded within a distance of 60 cm from the magnetic source and in a wide range of measurement environments, including a motion laboratory, treatment room or home situation (van Kordelaar et al. 2012).

#### Paradigm and data analysis

One table with a height of 76 cm was used for all 3D kinematic measurements. While seated at this table, participants performed a functional reaching task. During this task, patients had to reach forward with the paretic arm to grasp a block (5 × 5 × 5 cm and 150 g) at maximum reaching distance. After picking up the block, they had to transport it to a target location, which was located at the contralateral side at a distance equal to the reaching distance. Patients were instructed not to slide their hand over the table and to perform the task at a comfortable pace. Seven trials were performed in each measurement. Details of the kinematic data acquisition and reach-to-grasp paradigm have been published elsewhere (van Kordelaar et al. 2012).

The start of reach-to-grasp was defined as the moment at which the forearm sensor exceeded 5 % of the maximum speed during the forward reach. The end of reach-to-grasp was defined as the moment at which the transportation of the block started and the block lost contact with the table. This moment was identified as the moment at which the forearm sensor exceeded 5 % of the maximum speed during the transportation of the block towards the target location. The time series for grip aperture were calculated from the start to the moment the block lost contact with the table and were filtered with a second-order Butterworth low-pass filter with a cut-off frequency of 20 Hz. All kinematic data processing was performed using custom-made programs in MATLAB version R2006a.

Movement duration was defined as the time between the start and end of reach-to-grasp. The smoothness of the grasp movement was quantified by the normalized jerk of the grasp aperture between the thumb and index finger (NJ_grasp_). NJ_grasp_ was calculated for each trial. NJ_grasp_ represents the smoothness of the grasp aperture signal and is defined as the amount of jerk (i.e. third derivative) in the grasp aperture signal, normalized for movement duration and net change in grasp aperture during the reach-to-grasp movement (Hogan and Sternad 2009). Specifically, normalized jerk was calculated as follows:

1 $$ \text{NJ}_{{\text{grasp}}} = \sqrt {\frac{1}{2}\int\limits_{{t_{{\text{start}}} }}^{{t_{{\text{end}}} }} {\text{jerk}_{{\text{grasp}}}^{2} (t)\text{d}t*\text{MD}^{5} /L_{{\text{grasp}}}^{2} } } , $$

where NJ_grasp_ represents the normalized jerk of the grasp aperture; *t*_start_ represents the time of the reach-to-grasp movement started; *t*_end_ represents the time at which the reach-to-grasp movement ended; jerk_grasp_ represents the third time derivative of the grasp aperture; MD represents the movement duration; and *L*_grasp_ represents the difference in grasp aperture between the start and end of reach-to-grasp. NJ is mathematically independent of movement duration and the net change in grasp aperture, as a result of the normalization of MD^5^/*L*^2^ (Hogan and Sternad 2009).

Details of the kinematic data analysis have been published elsewhere (van Kordelaar et al. 2014).

### Statistics

The change in the ARAT, FMA and %NHPT between week 6 and week 29 was assessed using two-sided paired samples *t* tests (*p* < 0.05).

Differences in ROI activation levels between weeks 6 and 29 were tested with repeated measures analysis of variance (ANOVA), with ROI (12 levels) and time of measurement (2 levels) as within-subject factors. Furthermore, a voxelwise analysis was performed to test for possible differences outside the predefined ROIs. Voxelwise differences in the activation maps between weeks 6 and 29 were estimated with a paired samples *t* test in SPM5. The resulting statistical maps were thresholded at *p* < 0.05 [family-wise error (FWE)-corrected].

We plotted the frequency distribution of the clinical data and NJ_grasp_ to check whether NJ_grasp_ was normally distributed. The change in MD and NJ_grasp_ between weeks 6 and 29 after stroke was assessed using paired *t* tests (two-sided, *p* < 0.05).

Repeated measures ANOVAs in SPSS (version 20.0, IBM Corporation, New York) were conducted to investigate the interaction between activation levels in the 12 ROIs and NJ_grasp_ at weeks 6 and 29 after stroke. In each ANOVA, activation levels in the 12 ROIs at weeks 6 or 29 were used as the within-subject factor, whereas NJ_grasp_ at weeks 6 or 29 was taken as a between-subject covariate. The interaction between activation in the ROIs and NJ_grasp_ specified whether activation in the ROIs was related to NJ_grasp_. The significance of the interaction was assessed using a Bonferroni correction to correct for multiple testing, resulting in a significance level of *p* < 0.05/4 = 0.01. In case of a significant interaction between activation levels and NJ_grasp_, separate Pearson correlation coefficients were calculated between each ROI and NJ_grasp_. In addition, Pearson correlation coefficients were used to assess whether there was a mutual relationship between NJ_grasp_ and basic 3D kinematic and clinical measures including MD, ARAT, FMA and NHPT. The significance level for these post hoc correlation tests was set conservatively at *p* < 0.01 (two-sided) in order to avoid a type I error as a result of multiple testing.

## Results

Table 1 shows the characteristics of the patients included in this study. The patients improved significantly from week 6 to week 29 as assessed with the FMA (*t* = −2.911, *p* = 0.010), ARAT (*t* = −2.748, *p* = 0.014) and %NHPT (*t* = −6.044, *p* = 0.000). The ‘Appendix’ shows that thirteen patients had subcortical infarctions in the capsular region, whereas in two patients the infarction extended into the cortex. Two patients had pontine ischaemic infarctions. No infarcts included the primary motor cortex (Brodmann area 4). The average (±SD) time poststroke at which the first fMRI measurement took place was 6.4 ± 2.1 weeks and 5.9 ± 1.1 weeks for the kinematic assessment. The second session took place at 29.4 ± 4.7 weeks after stroke for fMRI and 28.8 ± 1.2 weeks for kinematic assessment.

**Table 1:** Patient characteristics at 6 and 29 weeks after stroke

Patient	Age (years)	Gender	Hand	Hem	Location	FMA W6	FMA W29	ARAT W6	ARAT W29	%NHPT W6	%NHPT W29	MD W6	MD W29	NJ_grasp_ W6	NJ_grasp_ W29
1	73	M	R	R	C	63	60	32	34	6	14	1.35	1.11	5.37	4.23
2	63	M	R	R	SC	51	65	39	57	21	66	5.08	1.34	3.70	3.45
3	60	M	R	R	C	57	60	36	40	7	43	2.68	1.83	4.21	3.66
4	66	M	R	R	SC	63	61	57	57	48	51	1.07	0.99	3.82	3.46
5	45	M	R	R	P	14	58	4	56	0	58	1.12	1.01	5.08	3.63
6	32	M	R	R	SC	44	57	47	55	0	46	1.14	1.11	3.49	3.37
7	71	M	R	L	P	65	66	57	57	101	103	1.29	1.41	3.65	3.60
8	64	M	L	R	SC	62	63	52	57	58	72	2.79	1.54	3.41	3.51
9	37	F	R	R	SC	59	63	47	57	40	67	2.33	1.09	4.06	3.77
10	65	M	R	R	SC	63	63	57	57	55	54	1.61	1.23	4.31	3.68
11	65	M	R	L	SC	46	54	31	37	28	72	1.07	0.81	3.48	3.36
12	54	M	R	R	SC	44	57	31	38	0	46	1.45	1.37	4.57	4.03
13	79	F	R	L	SC	66	66	57	57	80	96	1.60	0.95	3.43	3.89
14	73	M	R	L	SC	46	61	22	57	0	59	1.96	1.35	4.26	3.65
15	56	M	R	R	SC	56	66	36	57	26	82	1.28	0.97	3.96	3.79
16	57	F	R	R	SC	54	56	48	44	36	57	1.47	1.32	3.68	3.55
17	59	M	R	L	SC	62	65	48	57	48	82	1.07	0.79	3.66	3.41
Mean	59.9					55.0	61.6	41.20	51.4	32.5	62.8	1.79	1.19	4.00	3.65
SD	12.6					12.7	3.7	14.5	8.7	30.0	21.3				
Total		3 F/14 M	1L/16R	5L/12R	2P/2C/13SC										

M, male; F, female; Hand, handedness; R, right; L, left; Hem, lesional hemisphere, FMA, upper limb section of the Fugl-Meyer motor assessment; ARAT, Action Research Arm Test; NHPT, nine-hole peg test; MD, movement duration in seconds; NJ_grasp_, log values of normalized jerk between the thumb and index finger; P, pontine; C, extending to cortex; SC, subcortical; Y, years

* NHPT results are given as a percentage of norm scores (corrected for age and handedness)

**Fig. 1:**
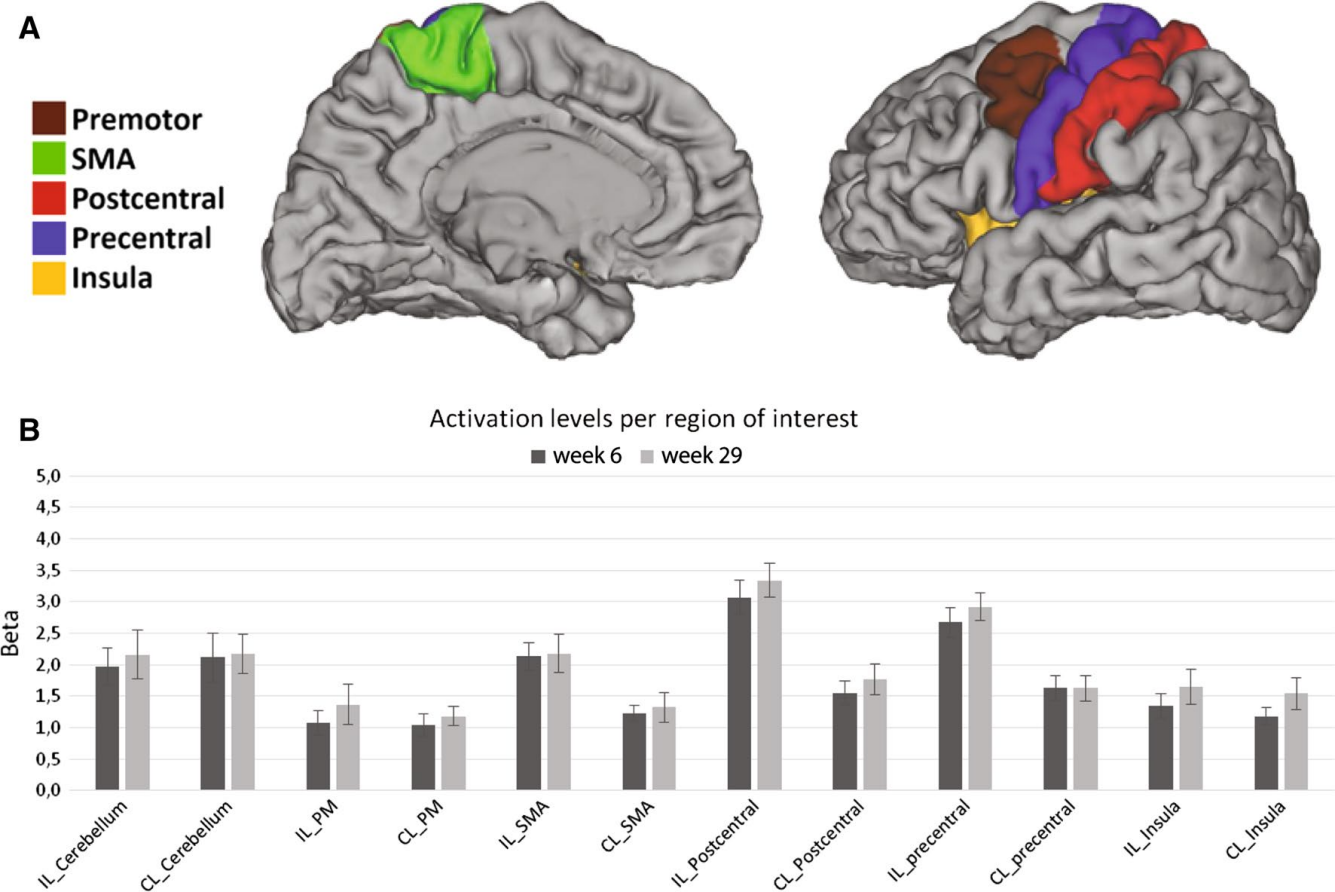
**a** Example of definition of cortical ROIs for one patient. **b** Mean results for task-related activity for the affected hand at weeks 6 and 29 after stroke. Mean beta values (±1 SE) in the cerebellum, premotor area (PM), supplementary motor area (SMA), postcentral gyrus, precentral gyrus and insula for the left (affected) and right (unaffected) hemispheres (LH and RH, respectively). Patients’ T-maps were flipped so the affected hand corresponded to the right hand

Activation in all ROIs was not significantly different between week 6 and week 29 (*F* = 0.699, *p* = 0.415) (Fig. 1). We checked whether this lack of significant results could be caused by variations in the quantity of mirror movements between sessions. However, all correlations between EMG score and ROI activation were not significant (all correlations had *p* > 0.085) for subjects with successful EMG measurements (week 6, *N* = 13 and week 29, *N* = 9). Problems with the acquisition hardware resulted in the absence of EMG data for 4 subjects at week 6 and 7 subjects at week 29.

The analysis of the main effect of the flexion–extension task vs rest revealed activation in a broad network of motor areas during both sessions at week 6 and week 29. Voxelwise comparisons between the sessions at 6 and 29 weeks did not reveal any significant change in activation.

**Fig. 2:**
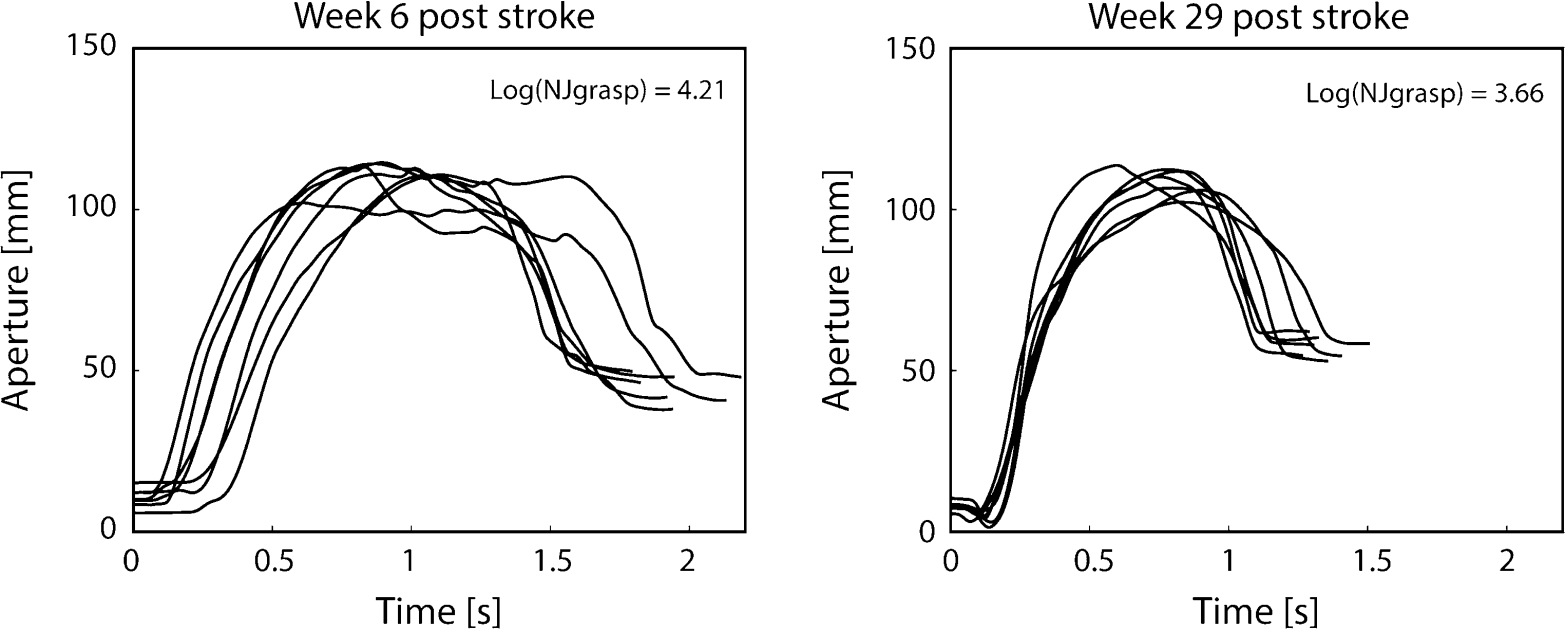
Grasp aperture between the thumb and index finger during the reach-to-grasp movement for one patient with stroke at weeks 6 and 29 after stroke. Each line represents one repetition of the task. Log(NJ_grasp_) values for this patient are provided for weeks 6 and 29 after stroke

NJ_grasp_ values were log-transformed, to meet assumptions of normality. The mean log(NJ_grasp_) values were 4.00 (SD = 0.57) and 3.65 (SD = 0.24) in week 6 and week 29, respectively. The hand aperture traces of a patient that showed log(NJ_grasp_) values close to the group mean values are shown in Fig. 2. A paired *t* test showed a significant decrease in log(NJ_grasp_) (*t* = 3.3, *p* = 0.004) and MD (*t* = 2.72, *p* = 0.015) between weeks 6 and 29.

**Fig. 3:**
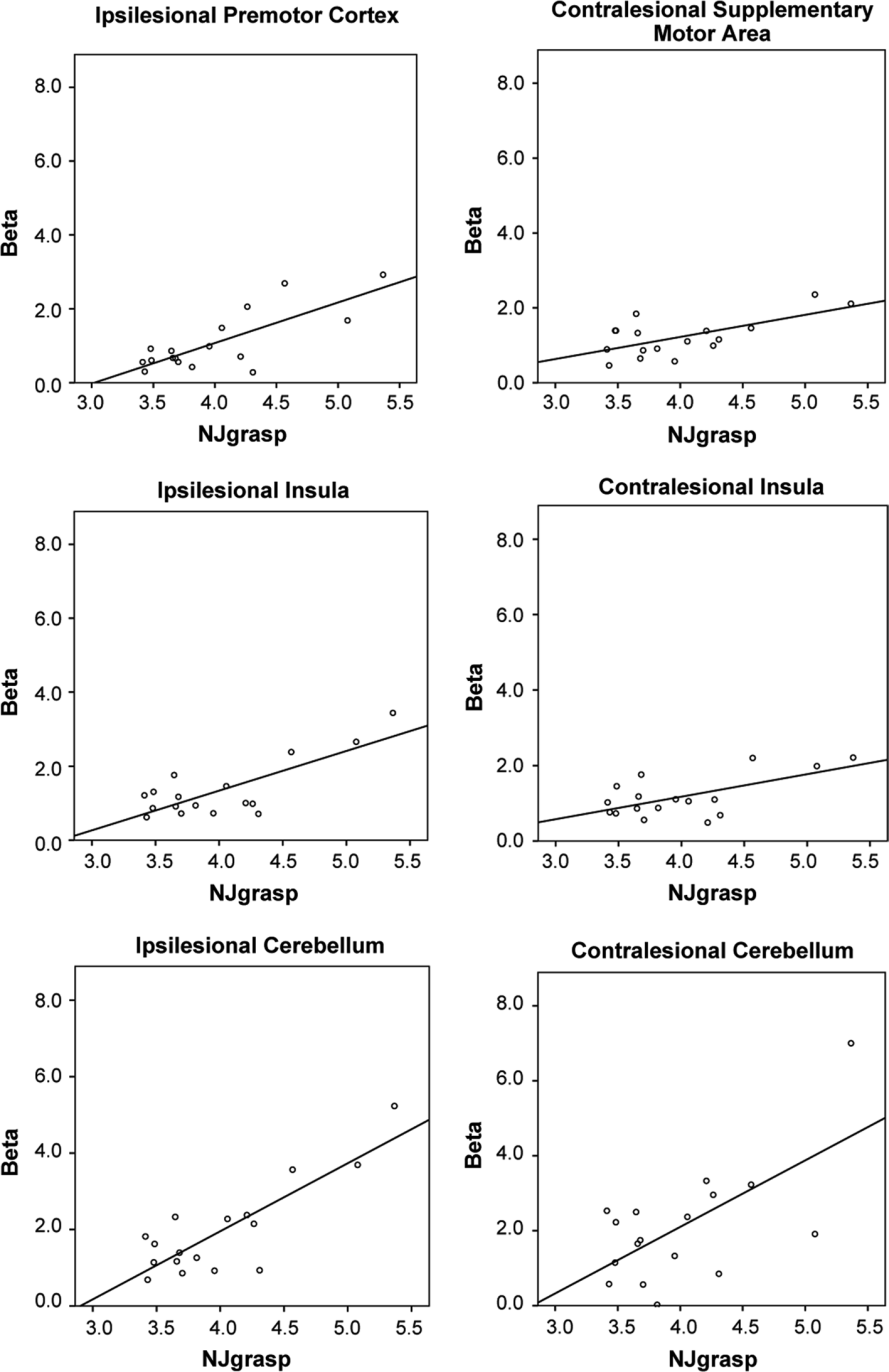
Scatterplots with regression line of significant correlations between beta values of individual ROIs and log values of NJ_grasp_

Table 2 shows that task-related activation in the various ROIs at week 6 after stroke interacted significantly with log(NJ_grasp_) at week 6 after stroke. Results from the other three ANOVAs were not significant after Bonferroni correction. However, a positive trend towards an interaction between activation in various ROIs and log(NJ_grasp_) was observed at week 29. Pearson correlations showed that increased activation in the ipsilesional premotor cortex, insula and cerebellum and the contralesional supplementary motor area, insula and cerebellum was significantly (*p* < 0.01) and positively associated with log(NJ_grasp_) at week 6 (Table 3). The significant correlations between activation in ROIs and NJ_grasp_ are also shown by the scatterplots in Fig. 3. Almost all ROIs that showed significant correlation with NJ_grasp_ also showed a significant correlation with MD, except for the contralesional cerebellum. The activation level in the contralesional precentral gyrus was significantly correlated with MD but not with NJ_grasp_. In addition, one negative correlation was found between ARAT scores and brain activation in the ipsilesional premotor cortex at week 6, indicating that poor upper limb capacity was correlated with increased activation of this ROI. No significant correlations were found between the ROI activation levels and the FMA scores at week 6. Lastly, log(NJ_grasp_) was significantly related to ARAT (*R* = −0.635, *p* < 0.001) and MD (0.828, *p* < 0.001) at week 6. No significant relation was found between Log(NJ_grasp_) and FMA (*R* = −0.381, *p* = 0.131) and NHPT (*R* = −0.542, *p* = 0.025).

**Table 2:** *F* values and significance levels for each combination of activation levels beta (within-subject factor) and NJ_grasp_ (between-subject covariate) at weeks 6 and 29 after stroke

	Beta week 6	Beta week 29
NJ_grasp_, week 6	*F* = 5.287, *p* = 0.002*	*F* = 1.914, *p* = 0.099
NJ_grasp_, week 29	*F* = 3.209, *p* = 0.021	*F* = 2.669, *p* = 0.029

NJ_grasp_, normalized jerk of grasp aperture

* *p* < 0.01

**Table 3:** Post hoc Pearson correlation coefficients (*R*) and significance levels (*P*) between each ROI and NJ_grasp_ at week 6 after stroke

	NJ_grasp_		MD		FMA		ARAT	
	*R*	P*	*R*	P*	*R*	P*	*R*	P*
I premotor cortex	0.776	*<0.001*	0.639	*0.006*	−0.336	0.188	−0.637	*0.006*
I supplementary motor area	0.316	0.216	0.359	0.157	−0.290	0.259	−0.397	0.115
I postcentral gyrus	−0.106	0.685	−0.049	0.851	0.069	0.793	0.187	0.473
I precentral gyrus	−0.019	0.943	0.057	0.829	−0.125	0.634	−0.100	0.703
I insula	0.778	*<0.001*	0.691	*0.002*	−0.340	0.182	−0.474	0.055
I cerebellum	0.832	*<0.001*	0.709	*<0.001*	−0.310	0.225	−0.538	0.026
C premotor cortex	0.380	0.133	0.374	0.139	−0.275	0.285	−0.313	0.221
C supplementary motor area	0.665	0.005	0.642	0.005	−0.458	0.065	−0.507	0.038
C postcentral gyrus	0.373	0.140	0.515	0.034	−0.468	0.058	−0.331	0.195
C precentral gyrus	0.486	0.048	0.608	*0.010*	−0.513	0.035	−0.450	0.070
C insula	0.617	*0.008*	0.621	*0.008*	−0.439	0.078	−0.463	0.061
C cerebellum	0.639	*0.006*	0.373	0.140	0.013	0.962	−0.336	0.187

For illustration purposes, we included bivariate correlation coefficients between activation levels in each ROI, movement duration, the upper limb section of the Fugl-Meyer motor assessment and the Action Research Arm Test

I, ipsilesional; C, contralesional; NJ_grasp_, log-transformed values of normalized jerk of the grasp movement; MD, movement duration; FMA, upper limb section of the Fugl-Meyer Motor Assessment; ARAT, Action Research Arm Test; R, Pearson correlation coefficient; P, significance value

* Significant correlations are italicized

## Discussion

The key finding of the present study was that jerkiness correlated highly and positively with levels of brain activity in the ipsilesional premotor cortex, insula and cerebellum and the contralesional supplementary motor area, insula and cerebellum at week 6 after stroke. This finding confirms part of our hypothesis that elevated recruitment of secondary sensorimotor areas would be associated with jerky movements.

Regarding effects of time, patients improved significantly on the clinical assessment scales including the ARAT (~10 points), FMA (~7 points) and %NHPT (~30 percentage points) from week 6 to week 29. The improvements exceeded the minimal clinically important differences of 5.7 points, 6.6 points and 10 % reported for the ARAT, FMA and %NHPT, respectively (Van der Lee et al. 1999), reflecting clinically relevant improvements between the two sessions. The reduction in movement duration between sessions was not significant although a trend was visible. In line with a previous longitudinal study, the quality of grasping control improved as reflected by a significant decrease in jerkiness of grasp aperture between weeks 6 and 29 after stroke (van Kordelaar et al. 2014). In this previous study, the greatest improvement occurred during the first 5 weeks after stroke and only a relatively small amount of improvement may have occurred between week 6 and week 29. This relatively minor improvement in motor control may explain why in the present study no significant change in brain activation patterns was observed between weeks 6 and 29 after stroke, neither with whole-brain analyses nor with ROI analysis.

In addition, the significant association between brain activation levels and smoothness was absent in week 29 after stroke. However, even after the Bonferroni correction, a positive trend towards an association between brain activation and jerk was still present at 29 weeks, suggesting that a significant association might be observed when sample size is increased.

Previous studies have already shown that activity in the contralesional hemisphere early after stroke is associated with reduced functional capacity as indicated by poor performance on clinical assessment scales (Buma et al. 2010; Ward et al. 2004). Moreover, focal activation in the ipsilesional hemisphere, contralateral to the moving hand as observed in healthy controls (Ward et al. 2003), is related to a favourable prognosis after stroke (Stinear 2010). The present study extends on this finding, showing that additionally recruited secondary sensorimotor areas are highly associated with jerky grasping movements in the subacute phase at 6 weeks after stroke.

The mechanisms underlying disruptions of smoothness are, however, poorly understood. After stroke, cortico-spinal pathways required for selective motor control are interrupted as shown with TMS (Stinear et al. 2007). This disrupted cortico-spinal control after stroke affects the execution of preplanned movements (Daly et al. 2006) and selecting the optimal ballistic movement strategy during functional tasks (Meulenbroek et al. 2001). As a consequence, patients must adapt their motor behaviour in order to compensate for these motor impairments. Given that jerk represents the change in acceleration (Rohrer et al. 2002), an increase in this metric may reflect the extent to which patients with stroke adjust their coordination patterns during a movement to correct for deviations from the intended movement pattern. This suggests that an increase in this metric reflects a type of control in which deviations from an optimal movement pattern are continuously corrected, possibly based on proprioceptive and visual feedback information. Therefore, the observed relationship between brain activation and smoothness, as quantified by jerk, suggests that secondary sensorimotor areas may be specifically involved in this error correction process.

In particular, the cerebellum is believed to play an important role in feedback-driven motor control and motor learning (Ramnani et al. 2001). In healthy subjects, ipsilateral and contralateral cerebellar activity has been found to be involved in closed-loop control during goal-directed upper limb movements using proprioceptive input and an internal copy of outgoing motor commands, i.e. efference copy (Ramnani et al. 2001). In stroke patients, the sensory motor representation of movements is likely disturbed and this representation must be relearned. The potential involvement of the cerebellum may highlight the interconnectedness between the cortex and cerebellum—a phenomenon yet to be fully understood. One would expect a higher demand on the cerebellum in relearning grasping or flexion–extension movements with the fingers in stroke (Hubbard et al. 2014). There is growing evidence that transfer of motor learning is accompanied with an increased reliance on the cerebellum (Seidler 2010; Dayan and Cohen 2011).

In addition, previous studies have shown that during finger movements the premotor cortex seems to be more involved in patients with stroke as compared to healthy subjects (Johansen-Berg et al. 2002) and is associated with a higher cognitive demand (Dennis et al. 2011). The present study suggests that this increased contribution of the premotor cortex does not necessarily improve quality of motor control. More generally, the present study suggests that a wide network of secondary sensorimotor areas may be involved in an adaptive relearning process in which stroke patients gradually regain the ability to reach for and grasp objects. Indeed, in a recent study Kantak and colleagues showed changes in the motor network after robotic reach training in healthy adults (Kantak et al. 2013).

### Scientific and clinical implications

The size and significance of the correlations between brain activation and normalized jerk were similar to the correlations between brain activation and movement duration. In addition, movement duration and normalized jerk were also strongly and negatively correlated, indicating that patients with jerkier movements took longer to complete the reach-to-grasp task. A mathematical relation between normalized jerk and movement duration can be ruled out as an explanation for this correlation as these variables are mathematically independent (Hogan and Sternad 2009). This finding therefore suggests that movement duration may directly depend on the brain’s capacity to control the quality of movement. This implication is supported by a previous study in which movement duration and normalized jerk showed the same longitudinal recovery pattern after stroke (Van Kordelaar et al. 2014).

Normalized jerk was also significantly and negatively correlated with the ARAT, suggesting that patients with jerkier grasping movements also had a reduced capacity to perform functional activities with the paretic upper limb. However, the positive correlation between brain activation and jerk as obtained with 3D kinematics was stronger compared to the negative correlation between brain activation and the FMA as well as with the ARAT. Together, these findings imply that the measure of jerk captured with 3D kinematics has an added value next to ordinal clinical scales which measure improvement at an activities level and do not take quality of movement into account (Alt Murphy et al. 2012; Levin et al. 2009). To investigate neural dynamics underlying stroke recovery, jerk may add to our understanding of the changes in brain activation dynamics when patients are relearning skills and improving motor control.

The relationship between brain activation and normalized jerk further suggests that additional recruitment of sensorimotor areas after stroke may not correspond to restitution of motor function, but more likely to adaptive motor learning strategies to compensate for motor impairments as reflected by an increase in jerk. Translational research programmes, such as EXPLICIT-stroke, should therefore establish whether therapies focusing on improving body functions, while avoiding compensation strategies, are able to promote restoration of neural networks in the cortex which may lead to improvements in quality of motor control (Kwakkel et al. 2008; van Vliet et al. 2013; Dobkin and Carmichael 2015).

To optimally benefit from this apparent added value of 3D kinematics, we argue that the development of motion trackers should be oriented to facilitating the use of 3D kinematics in clinical research. We have previously shown that we were able to use a mobile 3D kinematic set-up in order to realize an intensive follow-up of patients in the first 6 weeks and up to 6 months after stroke (Van Kordelaar et al. 2012). The advantage of the jerk measure as used in this study is that it can be obtained with only two kinematic sensors on the fingers and does not require full arm kinematics, which reduces donning time and hence improves clinical applicability of this measure. Moreover, low-cost cameras in combination with innovative motion tracker software can register 3D kinematics even without the need to attach markers or sensors to the body (Brokaw et al. 2013; Kurillo et al. 2013). These recent developments are highly promising with regard to the use of 3D kinematics in clinical research and clinical practice. We favour the implementation of these kinds of mobile motion trackers as well as easy-to-measure kinematic variables such as jerk to investigate quality of motor control after stroke.

### Limitations

Our findings should be considered in the context of the following limitations. First, as the included patients were generally mildly affected, the present results cannot be generalized to patients with a severe paresis of the upper limb, since severely affected patients were not able to perform the motor paradigms during the fMRI and 3D kinematic assessments. Second, the flexion–extension task that was administered in the scanner differed from the reaching task during the 3D kinematic measurements. Therefore, control strategies may have differed between the fMRI and 3D kinematic measurements. For instance, patients were able to rely on visual feedback during the 3D kinematic measurements, whereas this was not possible during fMRI scanning. Furthermore, we used a continuous and rhythmic task during fMRI scanning, whereas we used a discrete reaching task during 3D kinematic measurements. However, we argue that there is sufficient overlap, since the motor tasks during fMRI scanning and the reach-to-grasp task during the 3D kinematic assessments required flexion–extension of the fingers, which is considered an improvement with respect to the often used comparisons between fMRI and clinical tests. Third, given the large number of patients with a right hemispheric lesion (*N* = 12) compared to patients with a left-sided lesion (*N* = 5), possible effects of lesion side could not be investigated. Fourth, the measurements were performed at weeks 6 and 29 after stroke. Earlier fMRI scanning was impossible since patients were required to show sufficient finger extension to perform the motor paradigm. However, the moment of 6 weeks after stroke was well within the critical time window of 10 weeks after stroke in which most spontaneous neurological recovery is observed (Buma et al. 2013). Lastly, the fact that we found a relation between jerk and neural activation at week 6 but not at week 29 might be due to a lack of power, as we included a relatively small sample of 17 patients and variation in BOLD signal appeared to be considerable between and within subjects. In part, this variation between subjects may have been caused by differences in therapy as patients were allocated to different intervention groups within the EXPLICIT-stroke trial (Kwakkel et al. 2016). However, as severity of the initial motor impairment determines most of the variance in motor outcome between patients (Langhorne et al. 2011), we argue that differences in intervention would only have a minor effect on the variance between patients and the found correlations between smoothness and brain activation.

Future studies should therefore investigate correlations between brain activation patterns and quality of motor control, using large sample sizes, starting at an earlier time point after stroke and following up with intensively repeated measurements to capture the changes in these correlations over time after stroke. This relationship should preferably be measured directly in real time, with, for example, EEG or TMS coupled with kinematic measurement.

## Figures and Tables

**Fig. 4:**
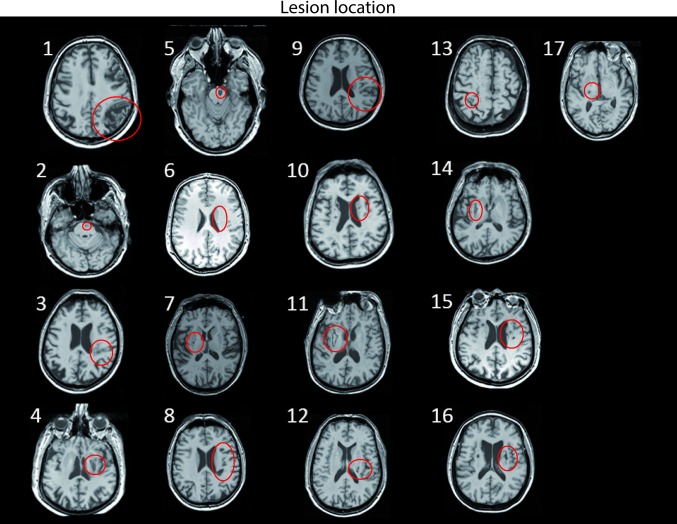
Axial structural T1-weighted MRI scans at the level of maximum infarct volume for each patient, obtained at the time of the second fMRI session at 6 months after stroke

## Appendix

For each patient the location of the lesion is indicated in Fig. 4.

## References

[CR1] Alt Murphy M, Willén C, Sunnerhagen KS (2012). Movement kinematics during a drinking task are associated with the activity capacity level after stroke. Neurorehabil Neural Repair.

[CR2] Brokaw EB, Lum PS, Cooper RA, Brewer BR (2013). Using the kinect to limit abnormal kinematics and compensation strategies during therapy with end effector robots. IEEE Int Conf Rehabil Robot.

[CR3] Buma FE, Lindeman E, Ramsey NF, Kwakkel G (2010). Functional neuroimaging studies of early upper limb recovery after stroke: a systematic review of the literature. Neurorehabil Neural Repair.

[CR4] Buma FE, Kwakkel G, Ramsey NF (2013). Understanding upper limb recovery after stroke. Restor Neurol Neurosci.

[CR5] Caimmi M, Carda S, Giovanzana C, Maini ES, Sabatini AM, Smania N, Molteni F (2008). Using kinematic analysis to evaluate constraint-induced movement therapy in chronic stroke patients. Neurorehabil Neural Repair.

[CR6] Daly JJ, Fang Y, Perepezko EM, Siemionow V, Yue GH (2006). Prolonged cognitive planning time, elevated cognitive effort, and relationship to coordination and motor control following stroke. IEEE Trans Neural Syst Rehabil Eng.

[CR7] Dayan E, Cohen LG (2011). Neuroplasticity subserving motor skill learning. Neuron.

[CR8] Dennis A, Bosnell R, Dawes H, Howells K, Cockburn J, Kischka U (2011). Cognitive context determines dorsal premotor cortical activity during hand movement in patients after stroke. Stroke.

[CR9] Dobkin BH, Carmichael ST (2015) The specific requirements of neural repair trials for stroke. Neurorehabil Neural Repair **[Epub ahead of print]** 10.1177/1545968315604400 PMC4786476 26359342

[CR10] Fischl B, van der Kouwe A, Destrieux C, Halgren E, Ségonne F, Salat DH (2004). Automatically parcellating the human cerebral cortex. Cereb Cortex.

[CR11] Folstein MF, Folstein SE, McHugh PR (1975). A practical method for grading the cognitive state of patients for the clinician. J Psychiatr Res.

[CR12] Friston KJ, Holmes AP, Worsley KJ, Poline J-P, Frith CD, Frackowiak RSJ (1995). Statistical parametric maps in functional imaging: a general linear approach. Hum Brain Mapp.

[CR13] Fugl-Meyer AR, Jääskö L, Leyman I, Olsson S, Steglind S (1975). The post-stroke hemiplegic patient 1. A method for evaluation of physical performance. Scand J Rehabil Med.

[CR14] Galea MP, Castiello U, Dalwood N (2001). Thumb invariance during prehension movement: effects of object orientation. NeuroReport.

[CR15] Hogan N, Sternad D (2009). Sensitivity of smoothness to movement duration, amplitude, and arrests. J Mot Behav.

[CR16] Hubbard IJ, Carey LM, Budd TW, Levi C, McElduff P, Hudson S, Bateman G, Parsons MW (2014). A randomized controlled trial of the effect of early upper-limb training on stroke recovery and brain activation. Neurorehabil Neural Repair.

[CR17] Johansen-Berg H, Rushworth MFS, Bogdanovic MD, Kischka U, Wimalaratna S, Matthews PM (2002). The role of ipsilateral premotor cortex in hand movement after stroke. Proc Natl Acad Sci USA.

[CR18] Kantak SS, Jones-Lush LM, Narayanan P, Judkins TN, Wittenberg GF (2013). Rapid plasticity of motor corticospinal system with robotic reach training. Neuroscience.

[CR19] Kurillo G, Han J, Obdržálek S, Yan P, Abresch R, Nicorici A (2013). Upper extremity reachable workspace evaluation with kinect. Stud Heal Technol Inf..

[CR20] Kwakkel G, Kollen BJ (2013). Predicting activities after stroke: what is clinically relevant?. Int J Stroke.

[CR21] Kwakkel G, Meskers CGM, van Wegen EEH, Lankhorst GJ, Geurts ACH, van Kuijk AA (2008). Impact of early applied upper limb stimulation: the EXPLICIT-stroke programme design. BMC Neurol.

[CR22] Kwakkel G, Winters C, van Wegen EEH, Nijland RHM, van Kuijk AA, Visser-Meily A et al (2016) Effect of unilateral upper limb training in two distinct prognostic groups early after stroke: the EXPLICIT-stroke randomized clinical trial. Neurorehabil Neural Repair. doi:10.1177/1545968315624784 10.1177/1545968315624784 26747128

[CR23] Langhorne P, Bernhardt J, Kwakkel G (2011). Stroke rehabilitation. Lancet.

[CR24] Levin MF, Kleim JA, Wolf SL (2009). What do motor ‘recovery’ and ‘compensation’ mean in patients following stroke?. Neurorehabil Neural Repair.

[CR25] Lyle RC (1981). A performance test for assessment of upper limb function in physical rehabilitation treatment and research. Int J Rehabil Res.

[CR26] Merdler T, Liebermann DG, Levin MF, Berman S (2013). Arm-plane representation of shoulder compensation during pointing movements in patients with stroke. J Electromyogr Kinesiol.

[CR27] Meulenbroek RGJ, Rosenbaum DA, Vaughan J (2001). Planning reaching and grasping movements: simulating reduced movement capabilities in spastic hemiparesis. Mot Control.

[CR28] Murphy T, Corbett D (2009). Plasticity during stroke recovery: from synapse to behaviour. Nat Rev Neurosci.

[CR29] Neggers SFW, Hermans EJ, Ramsey NF (2008). Enhanced sensitivity with fast three-dimensional blood–oxygen-level-dependent functional MRI: comparison of SENSE-PRESTO and 2D-EPI at 3 T. NMR Biomed.

[CR30] Nijland RH, van Wegen EE, Harmeling-van der Wel BC, Kwakkel G, Investigators EPOS (2010). Presence of finger extension and shoulder abduction within 72 hours after stroke predicts functional recovery: early prediction of functional outcome after stroke: the EPOS cohort study. Stroke.

[CR31] Oldfield RC (1971). The assessment and analysis of handedness: the Edinburgh inventory. Neuropsychologia.

[CR32] Oxford Grice K, Vogel KA, Le V, Mitchell A, Muniz S, Vollmer MA (2003). Adult norms for a commercially available Nine Hole Peg Test for finger dexterity. Am J Occup Ther.

[CR33] Raemaekers M, du Plessis S, Ramsey NF, Weusten JMH, Vink M (2012). Test–retest variability underlying fMRI measurements. Neuroimage.

[CR34] Ramnani N, Toni I, Passingham RE, Haggard P (2001). The cerebellum and parietal cortex play a specific role in coordination: a PET study. Neuroimage.

[CR35] Rohrer B, Fasoli SE, Krebs HI, Hughes R, Volpe B, Frontera WR (2002). Movement smoothness changes during stroke recovery. J Neurosci.

[CR36] Schepers VP, Visser-Meily AM, Ketelaar M, Lindeman E (2005). Prediction of social activity 1 year poststroke. Arch Phys Med Rehabil.

[CR37] Seidler RD (2010). Neural correlates of motor learning, transfer of learning, and learning to learn. Exerc Sport Sci Rev.

[CR38] Stinear CM (2010). Prediction of recovery of motor function after stroke. Lancet Neurol.

[CR39] Stinear CM, Barber PA, Smale PR, Coxon JP, Fleming MK, Byblow WD (2007). Functional potential in chronic stroke patients depends on corticospinal tract integrity. Brain.

[CR40] Van Kordelaar J, van Wegen EEH, Nijland RHM, de Groot JH, Meskers CGM, Harlaar J (2012). Assessing longitudinal change in coordination of the paretic upper limb using on-site 3-dimensional kinematic measurements. Phys Ther.

[CR41] Van Kordelaar J, van Wegen EEH, Nijland RHM, Daffertshofer A, Kwakkel G (2013). Understanding adaptive motor control of the paretic upper limb early poststroke: the EXPLICIT-stroke program. Neurorehabil Neural Repair.

[CR42] Van Kordelaar J, van Wegen E, Kwakkel G (2014). Impact of time on quality of motor control of the paretic upper limb after stroke. Arch Phys Med Rehabil.

[CR100] Van der Lee JH, Wagenaar RC, Lankhorst GJ, Vogelaar TW, Devillé WL, Bouter LM (1999). Forced use of the upper extremity in chronic stroke patients: results from a single blind randomized clinical trial. Stroke.

[CR43] Van Rootselaar A-F, Maurits NM, Renken R, Koelman JHTM, Hoogduin JM, Leenders KL (2008). Simultaneous EMG-functional MRI recordings can directly relate hyperkinetic movements to brain activity. Hum Brain Mapp.

[CR44] Van Vliet P, Pelton TA, Hollands KL, Carey L, Wing AM (2013). Neuroscience findings on coordination of reaching to grasp an object: implications for research. Neurorehabil Neural Repair.

[CR45] Ward NS, Brown MM, Thompson AJ, Frackowiak RSJ (2003). Neural correlates of outcome after stroke: a cross-sectional fMRI study. Brain.

[CR46] Ward NS, Brown MM, Thompson AJ, Richard S, Frackowiak J (2004). The influence of time after stroke on brain activations during a motor task. Ann Neurol.

[CR47] Worsley K, Friston K (1995). Analysis of fMRI time-series revisited-again. Neuroimage.

